# Sex differences in adult rat insulin and glucose responses to arginine: programming effects of neonatal separation, hypoxia, and hypothermia

**DOI:** 10.14814/phy2.12972

**Published:** 2016-09-23

**Authors:** Ashley L. Gehrand, Brian Hoeynck, Mack Jablonski, Cole Leonovicz, Risheng Ye, Philipp E. Scherer, Hershel Raff

**Affiliations:** ^1^ Endocrine Research LaboratoryAurora St. Luke's Medical CenterAurora Research InstituteMilwaukeeWisconsin; ^2^ Departments of Medicine, Surgery, and PhysiologyMedical College of WisconsinMilwaukeeWisconsin; ^3^ Touchstone Diabetes CenterDepartment of Internal MedicineUniversity of Texas Southwestern Medical CenterDallasTexas

**Keywords:** Body temperature, body weight, glucagon, isothermia, newborn, sexual dimorphism

## Abstract

Acute neonatal hypoxia, a common stressor, causes a spontaneous decrease in body temperature which may be protective. There is consensus that hypothermia should be prevented during acute hypoxia in the human neonate; however, this may be an additional stress with negative consequences. We hypothesize that maintaining body temperature during hypoxia in the first week of postnatal life alters the subsequent insulin, glucose, and glucagon secretion in adult rats. Rat pups were separated from their dam daily from postnatal days (PD) 2–6 for the following 90 min experimental treatments: (1) normoxic separation (control), (2) hypoxia (8% O_2_) allowing spontaneous hypothermia, (3) normoxic hypothermia with external cold, and (4) exposure to 8% O_2_ while maintaining body temperature using external heat. An additional normoxic non‐separated control group was performed to determine if separation per se changed the adult phenotype. Plasma insulin, glucose, and glucagon responses to arginine stimulation were evaluated from PD105 to PD133. Maternal separation (compared to non‐separated neonates) had more pronounced effects on the adult response to arginine compared to the hypoxic, hypothermic, and hypoxic‐isothermic neonatal treatments. Adult males exposed to neonatal maternal separation had augmented insulin and glucose responses to arginine compared to unseparated controls. Additionally, neonatal treatment had a significant effect on body weight gain; adults exposed to neonatal maternal separation were significantly heavier. Female adults had significantly smaller insulin and glucose responses to arginine regardless of neonatal treatment. Neonatal maternal separation during the first week of life significantly altered adult beta‐cell function in a sexually dimorphic manner.

## Introduction

Acute hypoxia is a common neonatal stressor in premature infants (Martin et al. 1986; Frankel and Stevenson 1987; Miller and Martin 1992; Low et al. 1993); it can lead to spontaneous hypothermia and altered metabolism (Frappell et al. 1992; Clark and Fewell 1996; Wood and Gonzales 1996; Mortola 2004). The general consensus is that hypothermia should be prevented in the human neonate and that core body temperature should be maintained (Laptook and Jackson 2006; Watkinson 2006; Laptook and Watkinson 2008; Clifford and Hunt 2010). However, we and others have proposed that prevention of the spontaneous hypothermic response to hypoxia in the neonate may be detrimental (Clark and Fewell 1996; Wood and Gonzales 1996; Shankaran et al. 2005; Bruder et al. 2011; Guenther et al. 2012).

We have developed a rat model of neonatal hypoxia with and without prevention of spontaneous hypothermia as a model of human prematurity (Bruder et al. 2008a; Guenther et al. 2012). As such, we have characterized the spontaneous decrease in core body temperature in response to acute hypoxia (Bruder et al. 2011) and the effects of body temperature maintenance during acute hypoxia in postnatal day (PD) 2 and PD8 rat pups (Guenther et al. 2012). We have shown that 1‐h of continuous hypoxia while maintaining isothermia causes hyperinsulinemia in PD2 pups despite no change in plasma glucose. Based on these previous findings, it seems possible that applying external heat during hypoxic episodes to prevent hypothermia may be eliciting additional stress on the neonate and may possibly result in a disruption of glucose homeostasis.

In order to study hypoxia in the neonate without altering maternal physiology due to exposure to hypoxia (Bruder et al. 2008b), the normoxic control group for our model separates the neonatal rat pups from their lactating dam (Bruder et al. 2008a; Guenther et al. 2012). Since premature human infants are usually separated from their parents for significant periods of time for medical care, maternal separation adds to the strength of this model of prematurity. Therefore, in this study, we evaluated the long‐term effects of neonatal separation (normoxic separation time control), acute hypoxia allowing spontaneous hypothermia, induced acute hypothermia per se, and acute hypoxia while preventing spontaneous hypothermia (isothermia) on the subsequent control of insulin, glucose, and glucagon in the adult male and female rat. In the process, we were also interested in the effects of acute, daily separation of the neonates from the nursing dams (needed as a normoxic control) compared to neonates who were not separated from their dams. We hypothesized that hypoxia while maintaining body temperature at control levels will result in altered insulin, glucose, and glucagon secretion as an effect of long‐term metabolic programming. In the process, we also evaluated the hypothesis that separation of the neonates from their lactating dams alters the adult control of pancreatic islet cell hormone secretion.

## Materials and Methods

### Animal treatments and experimental protocols

Federal guidelines (http://grants1/nih/gov/grants/olaw/references/phspol.htm) for use and care of laboratory animals were followed and the protocols were approved by the Institutional Animal Care and Use Committee of Aurora Health Care. Timed pregnant Sprague–Dawley rats (*N* = 10) at gestational day E18 were obtained and housed in a standardized environment (lights on 0600–1800 h) and provided a standard diet and water ad libitum at Aurora St. Luke's Medical Center. Dams were allowed to deliver normally and care for their pups without interruption until experimentation. A total of 110 pups were studied as described below.

#### Neonatal treatments

Rat pups of both sexes on postnatal day 2 (PD2) were randomly assigned to the following acute, daily experimental treatments: normoxia unseparated (Norm‐Unsep as a control for normoxic separation per se), normoxia separated (Norm‐Sep as a normoxic control for the following neonatal treatments), hypoxia (Hypoxia), hypothermia (Hypotherm), and hypoxia while maintaining isothermia (Hypoxia‐Isotherm). Norm‐Sep was established a priori as the separation control group for hypoxia, hypothermia, and hypoxia‐isothermia treatments. All neonatal treatments were performed once daily for 90 min (between 0830 and 1000 h) from PD2 to PD6. No bedding changes occurred during the week of experimentation. Bedding changes occurred once per week after PD6.

*Normoxia unseparated* pups (*N* = 23) were left undisturbed from PD2 to PD6. *Normoxia separated* pups (*N* = 22) were removed from the dam and placed into a chamber with bedding and a variable setting heating pad set on the lowest setting required to prevent hypothermia in pups separated from their lactating dams in a normoxic environment (Guenther et al. 2012). *Hypoxia* pups (*N* = 25) were removed from the dam and placed into a chamber with bedding and a variable setting heating pad set on low heat. Hypoxia was induced by decreasing the chamber inflow O_2_ concentration to 8% (balance nitrogen) as described in detail previously (Bruder et al. 2008a, 2011; Guenther et al. 2012). This leads to a transcutaneous O_2_ saturation of approximately 80% (Bruder et al. 2008a). Body temperature was allowed to spontaneously decrease during hypoxia and was measured in a sentinel pup as described previously (Guenther et al. 2012). Body temperature had spontaneously decreased to 23.9 ± 0.5°C (*n *=* *10) after 90 min of hypoxia. Pups were warmed to normal body temperature range of 32–34°C using a variable setting heating pad set on low before returning the pups to the nest. *Hypothermia* pups (*N* = 15) were removed from the dam and placed in a chamber with bedding on top of a cold plate (Model #AHP‐1200CPV; TECALAB, Chicago, IL) set between 24 and 27°C, which could be adjusted depending on body temperature. Body temperature was measured in a sentinel pup using RET‐30‐Iso rectal probes and a BAT‐12 digital thermometer connected to a SBT‐5 switchbox (Physitemp Instruments, Clifton, NJ), and was decreased to 25°C over 30 min and held at 25°C for the duration of the acute exposure by adjusting the temperature of the cold plate. After hypothermia was completed, body temperature was allowed to increase to a normal range (32–34°C) using a variable setting heating pad set on low before returning the pups to the nest. *Hypoxia‐isothermia* pups (*N* = 25) were treated identically as the hypoxia pups except that body temperature was maintained at 32°C with a heat plate (Model #AHP‐1200CPV; TECALAB) as described previously (Guenther et al. 2012).

Sentinel pups used for monitoring body temperature were not used in subsequent experiments. All rat pups were weighed on PD6 after experimentation before being returned to the nest. On PD22, all animals were weaned and housed by sex and treatment group with two to three animals per cage. Weaned animals were given a standard diet and water ad libitium and only handled during weekly bedding changes. Food and water intake was not monitored. Animals were weighed every 2 weeks at PD22, PD37, PD51, PD65, PD79, and PD93.

#### Adult experiments: intraperitoneal arginine stimulation tests

Intraperitoneal (ip) arginine stimulation tests were performed on adult rats age PD105 to PD133 as previously described (Ye et al. 2015). Arginine stimulation was chosen rather than glucose tolerance tests because our previous data suggested that glucose tolerance tests are not suitably subtle (Gehrand et al. 2015). Additionally, we were interested in measuring a glucagon response to arginine stimulation (Ye et al. 2015). Rats were fasted for 16 h overnight before ip arginine testing. Blood (approximately 200 *μ*L) was collected via tail clip to establish a baseline sample (0 min) as described previously (Waner and Nyska 1994). l‐arginine monohydrochloride (Sigma A4599, St. Louis, MO) was diluted in isotonic saline and administered ip at 1 mg/kg after the baseline collection. Then, blood was collected via tail clip 5, 15, and 30 min post injection. Blood was processed for plasma and stored at −20°C until subsequent analysis.

### Insulin, glucose, and glucagon assays

Plasma insulin (Crystal Chem, Downers Grove, IL) was measured by ELISA and plasma glucose was measured spectrophotometrically using the glucose‐oxidase method (Pointe Scientific, Canton, MI) as described previously (Guenther et al. 2012). The plasma glucose assay does not detect arginine at concentrations up to 250 mg/mL. Plasma glucagon (Mercodia catalog # 10‐1281‐01; Winston Salem, NC) was measured by ELISA. The intra‐assay variability is 10.1–17.9%, the inter‐assay variability is 5.1–6.9%, and the sensitivity is 5.2 pg/mL. There is cross reactivity with rat glicentin (4.0%), and human/rat/mouse oxyntomodulin (2.0%). Body weight, plasma glucose, plasma insulin, and plasma glucagon were analyzed by two‐way ANOVA for repeated measures (SigmaPlot 12.5; Systat Software, Inc., San Jose, CA) and post hoc multiple comparisons by Holm–Sidak. Area under the curve (AUC) for plasma insulin, glucose, and glucagon was calculated for each rat individually using trapezoidal rule (SigmaPlot 12.5) prior to statistical analysis. AUCs were analyzed by two‐way ANOVA, *t*‐test, and post hoc multiple comparisons by Holm–Sidak. All data are expressed as mean ± SE and were normally distributed.

### Pancreata immunohistochemistry

Immediately after arginine stimulation testing, a subset of pancreata were removed and fixed overnight at room temperature in 10% formalin. The pancreata were washed three times in 75% ethanol, and stored in 75% ethanol at 4°C until analysis. The pancreata were then processed to paraffin sections and subjected to immunofluorescence of insulin and glucagon as previously described (Ye et al. 2014). The immunofluorescence signals from the whole sections were scanned into images on a Hamamatsu (Hamamatsu Photonics K.K., Bridgewater, NJ) Nanozoomer Digital Slide Scanner (20× mode) by the University of Texas Southwestern Medical Center Whole Brain Microscopy Facility. On the images from each individual rat, the areas of insulin and glucagon signals were quantitated, respectively, with ImageJ (National Institutes of Health, Bethesda, MD), and normalized against the total pancreas area. Two‐tailed student's *t*‐test was applied for pairwise comparisons.

## Results

### Effect of neonatal treatments on body weight

**Figure 1:**
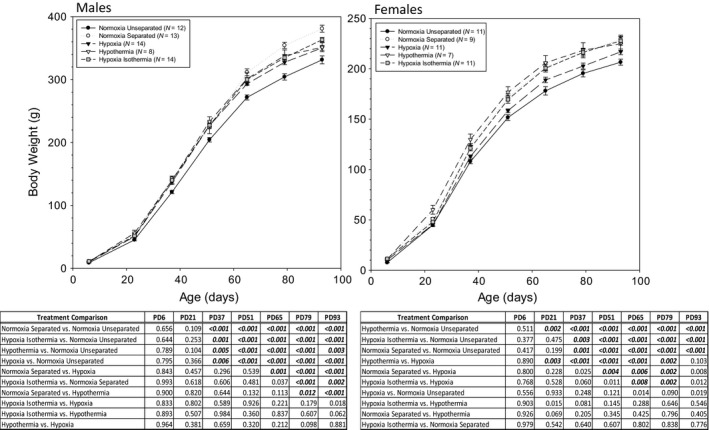
(A) Male and female body weights over time from PD6 to PD93. Rats were weighed at the completion of each treatment (PD6) and every 2 weeks post‐weaning until PD93. (B) Significant differences (*P* values) between neonatal treatments within gender are listed in the corresponding tables. Data are presented as mean ± SE. *N* values are shown in the figure labels.

All animals were weighed at PD6 (at the conclusion of all neonatal experimental treatments), at weaning, and then every 2 weeks until PD93 (Fig. 1). All animals gained weight in a similar pattern from PD6 to PD93. In males, there was no significant difference in body weight between treatments until PD37. In females, significant differences in body weight were observed starting at PD21. In both genders, the Norm‐Unsep group had the lowest body weight over time. Norm‐Sep males consistently had the highest body weight over time, and by PD93, the Norm‐Sep male body weight (380.9 ± 5.5 g, *n *=* *12) was significantly different from every other treatment (Norm‐Unsep = 331.4 ± 6.3 g, *n *=* *13; Hypoxia = 350.4 ± 3.8 g, *n *=* *14; Hypotherm = 351.4 ± 7.1 g, *n *=* *8; and Hypoxia‐Isotherm = 363.3 ± 3.6 g, *n *=* *14). In females, the Hypotherm group maintained the highest body weight over time (PD21 to PD79). At PD93, Norm‐Unsep females weighed significantly less than Norm‐Sep, Hypotherm, and Hypoxia‐Isotherm neonatal treatments (206.5 ± 3.0 g, *n *=* *11; 229.1 ± 4.2 g, *n *=* *9; 224.9 ± 6.6 g, *n *=* *6.6; and 227.8 ± 4.4 g, *n *=* *11, respectively).

### Arginine stimulation testing in adults after different neonatal treatments

**Figure 2:**
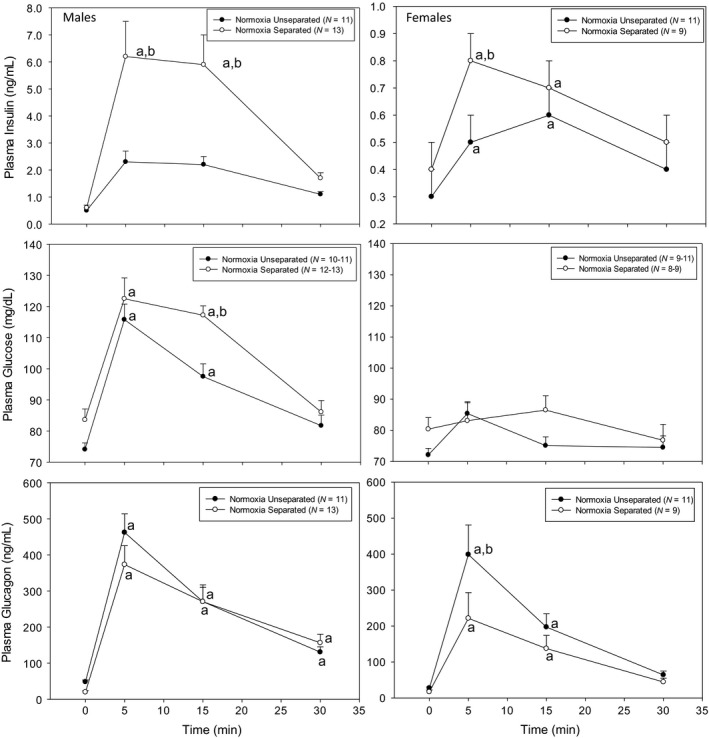
Intraperitoneal arginine stimulation test in adult rats previously exposed to neonatal normoxia separated and normoxia unseparated treatments. Plasma glucose, insulin, and glucagon were measured at pre‐arginine injection (0 min) and at 5, 15, and 30 min after arginine injection (1 mg/kg ip). ^a^Significantly different from baseline (0 min) within each neonatal treatment group. ^b^Significantly different from normoxia unseparated at same time point. Data are presented as mean ± SE. *N* values are shown in the figure labels.

**Figure 3:**
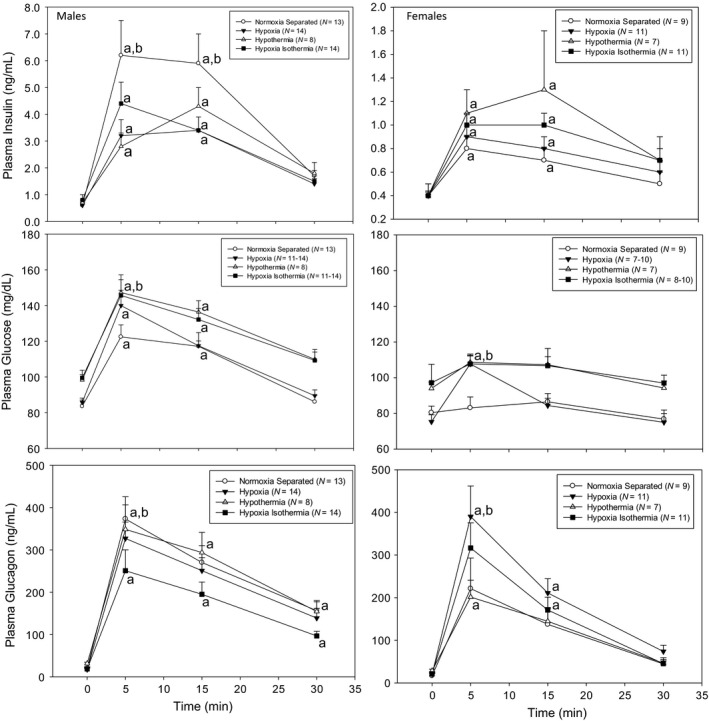
Intraperitoneal arginine stimulation test in adult rats previously exposed to neonatal normoxia separated, hypoxia, hypothermia, and hypoxia isothermia males and females. Plasma glucose, insulin, and glucagon were measured at pre‐arginine injection (0 min) and at 5, 15, and 30 min after arginine injection (1 mg/kg ip). ^a^Significantly different from baseline (0 min) within each neonatal treatment group. ^b^Significantly different from normoxia separated (control). Data are presented as mean ± SE. *N* values are shown in the figure labels.

**Figure 4:**
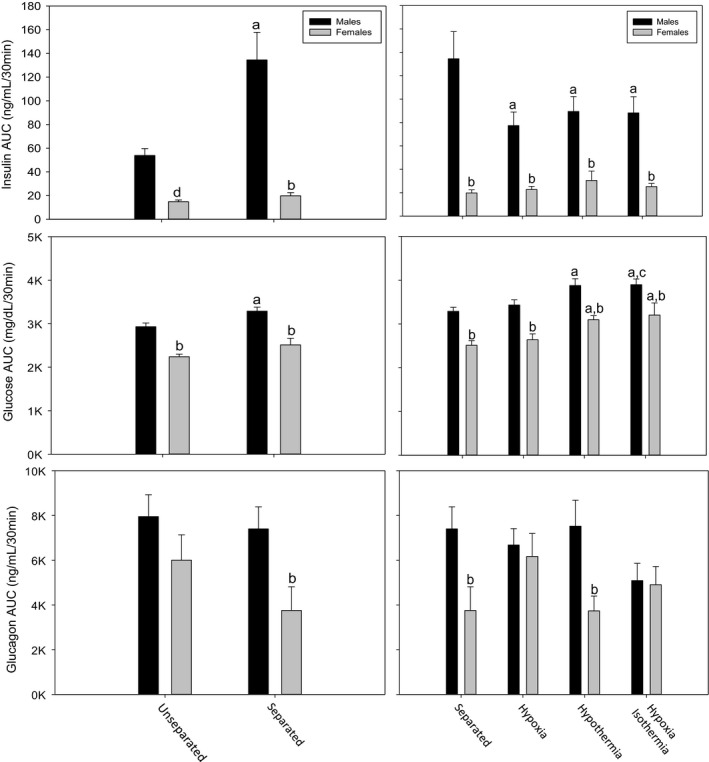
Integration of individual animal plasma insulin, glucose, and glucagon shown as areas under the curve (AUCs) during intraperitoneal arginine stimulation test in normoxia separated, normoxia unseparated, hypoxia, hypothermia, and hypoxia isothermia adult males and females. *Males:* normoxia unseparated (*N* = 12), normoxia separated (*N* = 13), hypoxia (*N* = 11), hypothermia (*N* = 8), hypoxia isothermia (*N* = 11). *Females:* normoxia unseparated (*N* = 11), normoxia separated (*N* = 9), hypoxia (*N* = 7), hypothermia (*N* = 7), hypoxia isothermia (*N* = 8). ^a^Significantly different from unseparated (left panel) and separated (right panel) within sex. ^b^Significantly different from male within treatment. ^c^Significantly different from hypoxia within males. ^d^Significantly different within treatment by *t*‐test. Data are presented as mean ± SE.

Plasma insulin, glucose, and glucagon levels during arginine stimulation testing are shown in Figures 2, 3, 4. Note that the *y*‐axis range is much smaller for female plasma insulin than male plasma insulin in Figures 2 and 3.

First, Norm‐Sep and Norm‐Unsep males and females were compared (Fig. 2 and Table 1). Within gender, female Norm‐Unsep plasma insulin (0.3 ± 0.1 ng/mL, *n *=* *11) and glucagon (27.5 ± 3.2 pg/mL, *n *=* *11) were significantly lower than Norm‐Unsep males (0.5 ± 0.1 ng/mL and 48.2 ± 3.2 pg/mL, *n *=* *12, respectively), while no differences were observed in Norm‐Sep treatment within gender (Table 1). Additionally, within males, Norm‐Sep plasma glucagon was significantly lower than Norm‐Unsep (19.8 ± 4.1 pg/mL, *n *=* *13, and 48.2 ± 3.2 pg/mL, *n *=* *12, respectively). Male Norm‐Sep plasma insulin levels at 5 and 15 min after arginine injection (6.3 ± 1.3 and 5.9 ± 1.1 ng/mL, *n *=* *13) were significantly higher than Norm‐Unsep males (2.3 ± 0.4 and 2.2 ± 0.3 ng/mL, *n *=* *11, respectively). Female Norm‐Sep plasma insulin levels were also significantly higher than Norm‐Unsep females at 5 min after arginine stimulation (0.8 ± 0.1 ng/mL, *n *=* *9, and 0.5 ± 0.1 ng/mL, *n *=* *11, respectively). The magnitude of the insulin response to arginine stimulation was much less in females than males. Male Norm‐Sep plasma glucose levels were significantly different than Norm‐Unsep at 15 min post arginine stimulation (117.2 ± 3.0 mg/dL, *n *=* *13 and 97.5 ± 4.1 mg/dL, *n *=* *12, respectively). No significant changes were observed in female plasma glucose levels over time in response to arginine stimulation. Plasma glucagon levels increased in both genders and both treatments (Norm‐Unsep and Norm‐Sep) after arginine injection. There was no significant difference in plasma glucagon levels in Norm‐Unsep and Norm‐Sep males in response to arginine stimulation. In females, Norm‐Unsep rats had a peak plasma glucagon level significantly higher than Norm‐Sep females at 5 min post arginine stimulation (398.8 ± 82.1 pg/mL, *n *=* *11 and 221.4 ± 71.6 pg/mL, *n *=* *9, respectively).

**Table 1:** Basal plasma insulin, glucose, and glucagon in adult rats

Neonatal treatment	Insulin (ng/mL)		Glucose (mg/dL)		Glucagon (pg/mL)	
	Male	Female	Male	Female	Male	Female
Normoxia unseparated	0.5 ± 0.1	0.3 ± 0.1^a^	74.1 ± 2.1	72.1 ± 2.1	48.2 ± 3.2	27.5 ± 3.2^b^
Normoxia separated	0.6 ± 0.1	0.4 ± 0.1	83.6 ± 3.5	80.4 ± 3.7	19.8 ± 4.1^b^	17.1 ± 5.1
Hypoxia	0.6 ± 0.1	0.4 ± 0.1	85.8 ± 2.4	75.3 ± 3.9	17.6 ± 3.2^b^	20.6 ± 3.5
Hypothermia	0.7 ± 0.1	0.4 ± 0.0^a^	98.3 ± 3.0^b^	94.0 ± 3.6^b^	31.4 ± 3.1	28.5 ± 3.8
Hypoxia isothermia	0.8 ± 0.2	0.4 ± 0.0^a^	99.5 ± 4.3^b^	97.2 ± 10.3^b^	18.6 ± 3.3^b^	21.3 ± 5.0

Data are mean ± SEM. For males: normoxia unseparated (*N* = 12), normoxia separated (*N* = 13), hypoxia (*N* = 14), hypothermia (*N* = 8), hypoxia isothermia (*N* = 14). For females: normoxia unseparated (*N* = 11), normoxia separated (*N* = 9), hypoxia (*N* = 11), hypothermia (*N* = 7), hypoxia isothermia (*N* = 11). Data are presented as mean ± SE.

a Female different from male within each row.

b Different from normoxia unseparated within each column.

Figure 3 shows the plasma insulin, glucose, and glucagon levels in Norm‐Sep, Hypoxia, Hypotherm, and Hypoxia‐Isotherm males and females in response to arginine stimulation. Plasma insulin was significantly increased in the Norm‐Sep males compared to the other treatment groups (Hypoxia, Hypotherm, and Hypoxia‐Isotherm) at 5 and 15 min post arginine stimulation. Otherwise, there were no differences between groups in males. There were no significant between‐group differences observed in female plasma insulin levels. Again, note that the magnitude of the plasma insulin response to arginine was much lower in the females compared to the males. Hypotherm and Hypoxia‐Isotherm males had significantly higher plasma glucose than norm‐sep males at 5 min post arginine stimulation (147.3 ± 9.9 mg/dL, *n *=* *8 mg/dL, 145.7 ± 8.8 mg/dL, *n *=* *12, and 112.5 ± 6.7 mg/dL, *n *=* *13, respectively). The magnitude of the plasma glucose response was less in females. At 5 min post arginine stimulation, plasma glucose in Norm‐Sep females (83.1 ± 6.1 mg/dL, *n *=* *9) was significantly lower than all other treatment groups (Hypotherm = 108.6 ± 4.7 mg/dL, *n *=* *7; Hypoxia = 107.7 ± 4.5 mg/dL, *n *=* *9; Hypoxia‐Isotherm = 107.7 ± 4.9 mg/dL, *n *=* *10). Only Hypoxia plasma glucose levels at 5 min post arginine stimulation were significantly different from baseline in females (107.7 ± 4.5 mg/dL, *n *=* *9 and 75.3 ± 3.9 mg/dL, *n = *10, respectively). Plasma glucagon levels were increased in response to arginine stimulation for all neonatal treatments and genders after arginine stimulation. In males, Norm‐Sep plasma glucagon levels were significantly higher than Hypoxia‐Isotherm at 5 min post arginine stimulation (373.4 ± 52.4 pg/mL, *n *=* *13 and 250.9 ± 49.5 pg/mL, *n *=* *14, respectively). In females, Hypoxia plasma glucagon levels were significantly higher than Norm‐Sep and Hypotherm at 5 min post arginine stimulation (390.3 ± 71.7 pg/mL, *n *=* *11; 221.4 ± 71.6 pg/mL, *n *=* *9; 201.4 ± 39.7 pg/mL, *n *=* *7, respectively).

In order to summarize the data in Figures 2 and 3, AUC analyses for the response to arginine were calculated for plasma insulin, glucose, and glucagon (Fig. 4). In most neonatal treatment groups, adult males had higher AUCs for insulin, glucose, and glucagon compared to adult females. By post hoc analysis after ANOVA, there was no significant difference between male and female plasma insulin AUC within Norm‐Unsep; however, the female insulin AUC was significantly lower by *t*‐test (*P *<* *0.001). Plasma insulin AUC for female Norm‐Sep was significantly lower than male Norm‐Sep. Within males, Norm‐Sep insulin AUC was significantly higher than Norm‐Unsep. In males, Hypoxia, Hypotherm, and Hypoxia‐Isotherm insulin AUCs were significantly lower than the control (Norm‐Sep). Plasma insulin AUCs of all groups (Norm‐Sep, Hypoxia, Hypotherm, and Hypoxia‐Isotherm) were significantly different between genders. There was no significant difference in plasma insulin AUC between treatments within females.

Male Norm‐Sep plasma glucose AUC was significantly higher than Norm‐Unsep. Female plasma glucose AUC was significantly lower than male plasma glucose AUC in Norm‐Unsep and Norm‐Sep treatments. Plasma glucose AUC in Hypotherm and Hypoxia‐Isotherm treatments were significantly higher than control (Norm‐Sep) in both genders. Additionally, Hypoxia‐Isotherm plasma glucose AUC was significantly higher than Hypoxia in males. Female plasma glucose AUC was significantly lower than males within each treatment (Norm‐Sep, Hypoxia, Hypotherm, and Hypoxia‐Isotherm). Female plasma glucagon AUC was significantly lower than male plasma glucagon AUC within Norm‐Sep and Hypotherm. No other significant differences were observed in plasma glucagon AUCs.

**Figure 5:**
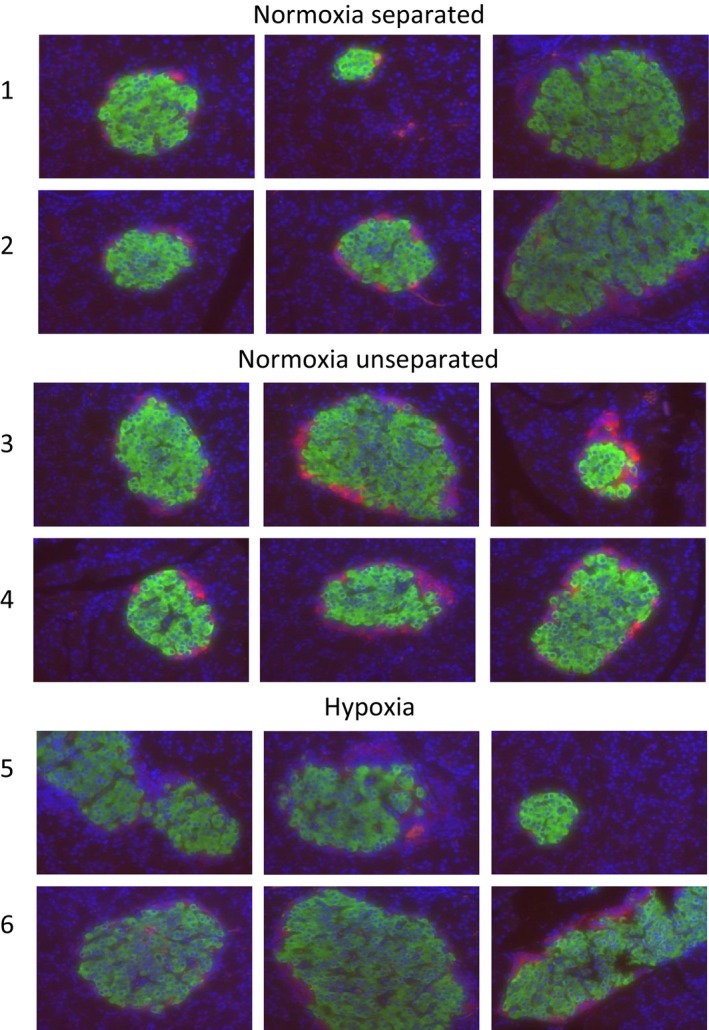
Immunofluorescence of insulin and glucagon in adult male pancreatic islets from the neonatal normoxia separated, normoxia unseparated, and hypoxia exposures. Pancreata were sectioned and stained for insulin (green), glucagon (red), and DAPI control (blue). Three individual islets from one pancreas per rat [1–6] are shown.

Randomly selected pancreata from adult male Norm‐Unsep, Norm‐Sep, and Hypoxia rats were analyzed for content of glucagon and insulin in the islets of Langerhans (Fig. 5). Each row designates three images from one rat pancreas. Overall, we did not observe significant differences in the glucagon (red) and insulin (green) staining between Norm‐Unsep, Norm‐Sep, and Hypoxia males. Total immunofluorescent quantification of glucagon and insulin analysis was performed on each of these individual slides. No significant differences in quantification of insulin or glucagon were observed (numerical data not shown).

## Discussion

We evaluated the effects of neonatal‐maternal separation, neonatal hypoxia allowing hypothermia, neonatal hypothermia per se, and neonatal hypoxia while maintaining body temperature (isothermia) on subsequent insulin, glucose, and glucagon responses to arginine in the adult male and female rat. Our major findings were: (1) Adult males separated from their dams daily during the first week of neonatal life had augmented insulin and glucose responses to arginine compared to adult males that were not separated as neonates. Additionally, adult males exposed to neonatal hypothermia and hypoxia while maintaining isothermia had significantly higher levels of plasma glucose at 5 min post arginine stimulation compared to adult males that were separated as neonates. (2) At 5 and 15 min after arginine stimulation, plasma insulin levels in adult males that had been separated from their dams as neonates was significantly higher than all other neonatal treatments (hypothermia, hypoxia, and hypoxia while maintaining isothermia). (3) The magnitude of insulin and glucose response to arginine was significantly less in adult females compared to adult males regardless of neonatal treatment. (4) Overall, adult males typically had higher levels of insulin, glucose, and glucagon compared to adult females. (5) Normoxic, unseparated neonates of either sex maintaining the lowest subsequent body weight gain over time. (6) Neonatal hypoxia while maintaining isothermia resulted in significantly higher plasma glucose levels in response to arginine stimulation in adult males compared to adult males exposed to neonatal hypoxia; there were no appreciable differences in plasma insulin or glucagon.

### Adult male insulin, glucose, and glucagon after neonatal separation

We found that the normoxic controls (pups separated from their dams daily) demonstrated augmented insulin and glucose, but not glucagon, responses to arginine in adults compared to adults not exposed to neonatal separation. Furthermore, basal (fasting) plasma glucose tended to be higher. Postnatal maternal separation has been linked to altered metabolic states later in life including decreased insulin secretion (Cook 1999; Mela et al. 2012), increased insulin secretion (Hofman et al. 1997), altered glucose homeostasis (McPherson et al. 2009), increased levels of glucocorticoids with subsequent decreased levels of growth hormone (Fish et al. 2004), and long‐lasting decreased levels of plasma leptin (Marco et al. 2015). It has been suggested by Mela et al. (2012) that there is an alteration in central leptin sensitivity as a result of an altered metabolic state in adult rats that had been exposed to maternal separation, since plasma leptin levels remained low throughout adulthood with no appreciable differences observed in hypothalamic mRNA levels for the leptin receptor (Mela et al. 2012). Additionally, Mela et al. found decreases in insulin levels in a sex‐dependent manner; however, no changes in insulin sensitivity were observed.

In this study, the augmented insulin and glucose responses to arginine in the adult males exposed to neonatal maternal separation suggests that (1) the insulin‐secreting beta cells of the pancreatic islets are hyper‐responsive despite no apparent change in islet insulin content and (2) that these adults may be more insulin resistant than the neonatally unseparated controls. The augmentation of the insulin response to arginine is possibly due to an increase in responsivity of the beta cells, and not due to an increase in the number of beta cells present in the pancreas, because we did not observe any differences in insulin content and beta cell number in the islets. It is also possible that there is augmented content of stored prepro‐ or proinsulin in the beta cells of the normoxic, separated males.

Of most interest in this study were the significant differences observed between the non‐separated and 90 min maternally separated groups. Maternal separation is a well‐known neonatal stress model developed to study acute and long‐term physiological and behavioral effects. However, maternal separation protocols can vary anywhere from 15 min to 5 h/day and span anywhere from birth to weaning at PD21 (McIntosh et al. 1999; Pryce et al. 2001; Kalinichev et al. 2002; Rees et al. 2006; Haley et al. 2013). Some protocols move the dam to a separate room to minimize ultrasonic communication from dam to pup (Foscolo et al. 2008), some separate the pups individually from the dam (Pryce et al. 2001), and some simply perform maternal separation for a single 24‐h period (Viveros et al. 2009; Mela et al. 2012; Clarke et al. 2013). Although brief periods of maternal separation of the dam from the pups in their nest is normal in the wild for rat species (Calhoun 1963), there does not seem to be a general consensus for how maternal separation should be performed in experimental animals. It is important to be aware that the maternal separation protocols bring into play other factors aside from just a separation stress. Separating the pup from the dam also includes altered maternal care upon dam‐pup reunion (Llorente et al. 2011), lack of proper nutrition (Viveros et al. 2009), and a decrease in body temperature due to lack of huddling with the dam. These different protocols should be thought of as a combination of all of these factors (Marco et al. 2015). We chose daily 90 min separation during PD2‐6 as a control for brief hypoxic exposures to model the hypoxia of prematurity due to lung disease (Cummings et al. 1989) and because this is within the parameters of normal rat behavior (Calhoun 1963).

### Hypoxia with spontaneous hypothermia, hypothermia, and isothermic hypoxia

The original motivation for this study was to investigate the long‐term effects of maintaining isothermia during acute hypoxia during the first week of neonatal life. We observed significantly higher plasma glucose levels in response to arginine stimulation in adult males exposed to neonatal isothermic hypoxia compared to adult males exposed to neonatal hypoxia without significant differences in plasma insulin or glucagon. Additionally, plasma glucose AUCs for adult females exposed to neonatal hypothermia and hypoxic isothermia were significantly higher than the normoxic separated control. We observed significant differences in plasma insulin, glucose, and glucagon in response to arginine stimulation between genders; however, there were no dramatic differences comparing hypoxia with spontaneous hypothermia, hypothermia, and hypoxia while maintaining control body temperature. We were, therefore, surprised that maintenance of body temperature during neonatal hypoxia did not have marked effects on subsequent adult insulin, glucose, and glucagon dynamics.

Although there are not a large number of studies in adults exposed to *postnatal* hypoxia in rats, there are numerous studies linking *fetal* hypoxia exposure to an increased risk of insulin resistance, changes in glucose metabolism, and diabetes development in the adult (Coughlan et al. 2004; Camm et al. 2011; Rueda‐Clausen et al. 2011; Cao et al. 2012). We have previously shown that neonatal acute hypoxia exposure with the maintenance of body temperature, increases plasma insulin and significantly alters the control of glucose in PD2 and PD8 rats (Guenther et al. 2012). Additionally, we have previously shown that neonatal *intermittent* hypoxia exposure acutely elicits hyperglycemia and hyperinsulinemia from PD2 to PD14 (Chintamaneni et al. 2013) and evokes changes in hypothalamic–pituitary–adrenal axis function and an upregulation of inflammatory‐related genes in the adult due to long‐lasting programming effects (Chintamaneni et al. 2014; Gehrand et al. 2015). Even though hypoxia leads to spontaneous hypothermia which is thought to be a protective mechanism against severe brain damage and metabolic stress in the neonate (Frappell et al. 1992; Clark and Fewell 1996; Steiner and Branco 2002; Ray et al. 2003), maintenance of isothermia in premature hypoxic human neonates is encouraged (Laptook and Jackson 2006; Watkinson 2006; Clifford and Hunt 2010). However, the long‐term effects of maintaining isothermia during acute but continuous hypoxic exposure are not fully studied. Our studies suggest that these effects are not particularly dramatic suggesting that acute neonatal *intermittent* hypoxia has significantly greater programming effects than acute *continuous* neonatal hypoxia (Chintamaneni et al. 2014).

Previously, we performed intraperitoneal glucose tolerance tests in male adult rats previously exposed to neonatal *intermittent* hypoxia and observed no difference in insulin, glucose, or C‐peptide from normoxic controls (Gehrand et al. 2015). Therefore, in this study, we used arginine stimulation testing as a more sensitive test to determine subtle differences in insulin dynamics (Ye et al. 2015) as arginine directly stimulates insulin release from beta cells in the pancreatic islets (Cherrington and Vranic 1973; Flatt and Bailey 1981; Larsson and Ahren 1998; Thams and Capito 1999). Another advantage of using arginine is its stimulation of glucagon secretion from the alpha cell of the islet (Cherrington et al. 1974; Gerich et al. 1974; Palmer et al. 1976; Ye et al. 2015).

### Males versus females

We observed a large (threefold) augmentation of the insulin response to arginine compared in adult males compared to females. It has previously been reported that neonatal maternal separation during the first 2 weeks of life typically causes hyperinsulinemia in the neonatal males, but not females (Viveros et al. 2009). Additionally, plasma glucose levels were not significantly different between sexes (Vital et al. 2006; Viveros et al. 2009). Haley et al. (2013) did not find significant differences in plasma glucose levels between sexes or treatments of non‐separated control, pups separated for 60 min/day, or pups separated for 60 min/day from PD5 to PD9; however, plasma insulin levels in adult males that were maternally separated for 60 min/day were significantly higher than their adult non‐separated counterparts. These differences were not observed in adult females. Interestingly, in another treatment group in Haley et al. (2013), mechanical and tactical stimulation of the pups provided during separation from the dam was able to prevent hyperinsulinemia in adult males. These findings suggest that consistent maternal care is necessary for a normal insulin response and a normal sensitivity to insulin.

The large increase in glucose in response to arginine in males compared to females was striking. Furthermore, the adult males in the neonatal separation group showed significantly higher plasma glucose responses compared to the unseparated neonates. The mechanism of the glucose response to arginine appears to be an early hepatic response to increased counterregulatory hormones like glucagon (Cherrington et al. 1974). The glucose remained elevated with the same increase in glucagon but a great insulin response is further evidence of a potential for insulin resistance in male adult rats who were separated daily from their dams during the first week of neonatal life. Others have demonstrated significantly lower insulin responses in adult female compared to male rats (Vital et al. 2006). Previous studies have shown that the mammalian hypothalamus is sexually dimorphic with male hypothalamic neurons developing earlier than females (Carrer and Cambiasso 2002; Sakuma 2009). It has been suggested that differential changes in hypothalamic development can be programmed through environmental changes, such as maternal separation, in a sex‐dependent manner, and thus induce differences in hypothalamic cell proliferation and maturation that affect downstream endocrine outcomes (Viveros et al. 2009). A possible mediator of this mechanism and important factor in normal development of specific hypothalamic circuits is leptin (Pinto et al. 2004), and it has been suggested that altered levels of leptin during important times of early development can result in an altered metabolic state later in life (McMillen et al. 2006). Viveros et al. (2009) demonstrated that maternal separation performed during a single 24‐h period at PD9, led to leptin levels that were nearly undetectable after 12 h of separation, and concluded that females were more sensitive to the effects of on the hypothalamus. That may be a mechanism involved in the dramatic male‐female differences we observed in our study.

### Body weight gain

The fetal insulin hypothesis aims to provide a link between low birth weight from malnutrition and beta cell dysfunction and type 2 diabetes mellitus (Hattersley and Tooke 1999). It was hypothesized that an epigenetic difference in beta cell dysfunction programmed from malnutrition can lead to a decrease in insulin sensitivity and altered whole‐body glucose metabolism in adulthood (Milner and Hill 1984). Low birth weight with subsequent rapid weight gain through adolescence in humans has been linked with poor glucose tolerance and insulin resistance (Crowther et al. 1998; Yajnik 2000) and this insulin resistance is thought to be programmed through undernutrition (Hales et al. 1991; Phillips 1998). Birth weight was not manipulated in our study; however, as expected, a difference in maternal care, including arched‐back nursing and excessive licking, was observed when the pups were returned to the dam after separation (Francis and Meaney 1999). This could cause a metabolic challenge by introducing different feeding patterns, as we observe significant differences in body weight between treatments.

All animals, regardless of neonatal treatment, gained weight in a similar pattern from PD6 to PD93. The neonatal normoxic unseparated treatment typically maintained the lowest subsequent body weight over time in males and females. The males who were separated from their dams as neonates maintained the greatest subsequent body weight gain from PD63 and older, while the females with neonatal hypothermic exposure maintained the greatest subsequent body weight gain from PD21 to PD79. Some studies have shown that neonatal‐maternal separation does not alter subsequent body weight gain compared to non‐separated controls (Cook 1999; Fish et al. 2004; McPherson et al. 2009; Haley et al. 2013). On the other hand, it has been suggested that maternal separated neonates are heavier than their control counterparts as adults (Slotten et al. 2006). Many have shown that maternal separation resulted in a reduction in body weight up to weaning at PD21 (Ogawa et al. 1994; McIntosh et al. 1999; Pryce et al. 2001; Kalinichev et al. 2002), and at PD45 (Foscolo et al. 2008). In our study, adults exposed to neonatal separation were significantly heavier compared to adults that were not separated, so it is possible that some degree of metabolic programming occurred during the first week of life resulting in increased weight gain.

Since food and water intake was not measured in our study, we cannot comment on whether the differences in body weights were due to a change in appetite or changes in metabolic programming from alterations in maternal care. In Clarke et al. (2013), pups in large litters (20 pups) showed a reduced growth rate that continued through adulthood compared to pups from a small litter (12 pups), and the body weight of pups from large litters were significantly lower than pups from a small litter by PD70. In this study, litter size did not exceed 12 pups.

Nursing rats in the wild will typically leave the nest for anywhere from 15 min to 3 h a day to gather food and water (Calhoun 1963). Additionally, differences in behavior in maternal care are observed when the pups are returned to the dam after an extended separation; among these are increased licking and grooming and engaging in arched‐back nursing (Francis and Meaney 1999). Dams that are never separated from their pups exhibit a more relaxed behavior (Francis and Meaney 1999). We did not observe any differences in body weight in males at weaning, but did in females. At weaning (PD21), females exposed to neonatal hypothermia were significantly heavier than hypoxic and normoxic unseparated females. This could be due to the dam supplying different maternal care between sexes.

We conclude that daily maternal separation for short periods of time during the first week of life alters the pancreatic beta cell response to arginine through metabolic programming and the effects of this are more pronounced in males than females. The data also suggest a sex‐specific effect of maternal separation on insulin sensitivity, although this will require specific experimental testing directed at this phenomenon. The cellular mechanisms for these findings are unknown, but we plan on investigating this further. Although we had hypothesized that adult rats exposed to neonatal hypoxia while maintaining isothermia would have an altered metabolic state compared to adults exposed to neonatal hypoxia, we did not find major effects of this treatment. We realize that this initial study did not evaluate potential mechanisms for the observed changes in insulin secretion and the potential for changes in insulin sensitivity. This will require perfecting a method to isolate pancreatic islet cells (Kelly et al. 2003) to evaluate potential molecular and electrophysiological changes in the adult due to neonatal treatment. Furthermore, studies to evaluate peripheral sensitivity to insulin are currently being planned. Although this study is descriptive and, in fact, the major finding of a dramatic effect of maternal separation was an unexpected byproduct of our interest in hypoxia, we hope that our findings stimulate others to study the phenomena described.

Maternal separation is now considered a model of early childhood stress in humans (Kuhn and Schanberg 1998; Faturi et al. 2010; Marco et al. 2015). Furthermore, because of the altricial nature of the rat (Romijn et al. 1991; Clancy et al. 2001, 2007; Callaghan et al. 2014), hypoxia and hypothermia in the first days of life in the rat can be considered a model of a common stress of prematurity (Sheldon et al. 1996; Huang et al. 2004; Watkinson 2006; Bruder et al. 2008a, 2011; Guenther et al. 2012). The major clinical implication of our study was that maternal care during the first week of life seems to be a major determinant of pancreatic islet cell function in adulthood and may also result in a decrease in peripheral insulin sensitivity. Of interest is that human beta cell populations are established preterm, and peak within the first 2 years of life (Gregg et al. 2012). Since our rat experiments model human prematurity, it may be that stress in the premature infant alters the development of the pancreatic islets, although we did not observe a significant change in islet cell composition. Finally, as alluded to above, preterm humans have a propensity for insulin resistance and the metabolic syndrome later in life, so it is possible that the early‐life stress of a premature birth contributes to this (Tinnion et al. 2014). Furthermore, our study adds to the growing concept of sex differences in the programming effects of neonatal stress.

## Conflict of Interest

The authors have no conflicts of interest to disclose.
